# Possibilities of Increasing the Durability of Punches Used in the Forging Process in Closed Dies of Valve Forgings by Using Alternative Materials from Tool Steels and Sintered Carbides

**DOI:** 10.3390/ma17020370

**Published:** 2024-01-11

**Authors:** Marek Hawryluk, Marta Janik, Zbigniew Gronostajski, Artur Barełkowki, Maciej Zwierzchowski, Marzena Lachowicz, Jacek Ziemba, Jan Marzec

**Affiliations:** 1 Department of Metal Forming, Welding and Metrology, Wroclaw University of Science and Technology, Lukasiewicza 5 Street, 50-370 Wroclaw, Poland; marta.janik@mahle.com (M.J.); zbigniew.gronostajski@pwr.edu.pl (Z.G.); artur.barelkowski@pwr.edu.pl (A.B.); maciej.zwierzchowski@pwr.edu.pl (M.Z.); marzena.lachowicz@pwr.edu.pl (M.L.); jacek.ziemba@pwr.edu.pl (J.Z.); jan.marzec@pwr.edu.pl (J.M.); 2 MAHLE Polska, Mahle 6, 63-700 Krotoszyn, Poland

**Keywords:** durability of forging tools, valve forging, chromium–nickel steel, methods of increasing tool life, sintered carbides

## Abstract

This study refers to an analysis of the durability of forging tools applied in the second operation of producing a valve forging from the chromium–nickel steel, NC3015. Due to the extreme working conditions of the tools, caused by cyclic thermo-mechanical loads, the average durability of tools made from tool steel WLV (1.2365) equals about 1500 forgings. An in-depth, complex analysis was performed on the technology, using macroscopic tests combined with a measurement of the wear/allowance on the tool working surface through 3D scanning; microstructural tests by means of light microscopy; observations of the changes taking place on the working surface with a scanning electron microscope; microhardness measurements; and multi-variant numerical simulations. It was established that the key issue is the proper selection of the process technological parameters, such as the input material and tool temperature, friction, lubrication, tribological parameters, type of tool material, or punch design, because even small changes made to them significantly affect the service life of forging punches. Therefore, to increase the durability of the forging dies, alternative materials made of W360, as well as two high speed steels, S600 and S705, were applied. However, the implemented punch materials did not bring the assumed effect of increased durability, as the highest average durability of steel W60 equaled only 1500 forgings, whereas in the case of the tool steels, this was below 900 forgings. For this reason, at the further stage, punches with sintered carbide inserts were introduced, which made it possible to significantly improve the durability up to the level of as many as 20,000 forgings, which, at the same time, points to a promising direction of further studies on the use of materials and solutions of this type.

## 1. Introduction

The continuous restrictions on combustion gas emissions cause fuel blends to have a poorer composition, which implies the need to improve the steels used for valves [1,2]. To prolong the operation time, steel producers have begun working on new compositions of steel used for valves, as well as new production technologies, with a special consideration for tool durability [3]. The valve construction, the operation environment, the type of the applied fuel blend, and the production have a significant effect on the durability of the valves [4]. Suction and exhaust valves have different constructions depending on the engine type [5]. They can be monolithic or bimetallic valves, monolithic valves with a welded valve foot, a hollowed mandrel filled with sodium to accelerate the cooling and increase the valve mass, or valves with reinforcement of the faying surface through plasma weld surfacing [6]. Exhaust valves are mostly bimetallic, with the following structure: an austenitic valve head and a martensitic mandrel. Suction valves are usually monolithic with a martensitic structure [7].

At present, we know of two technologies for producing valves for car engines [8,9]. The first is the resistance upsetting of a bar, followed by forging a valve and the extrusion and forging of a roller [10]. The upsetting process consists of locally heating the shank and then upsetting it to the required shape [11]. Valves made through the technology of upsetting and forging are non-homogeneous in their whole volume, which is connected with the point heating of the bar [12]. The main cause of defects formed in the upsetting process is overheating or lapping caused by too-high temperatures, improper preliminary beveling of the bar to a specific thickness, or the surface quality. Preparing the input material for the process is also costly; the bar should have the proper roughness, and there is a narrow tolerance for its diameter. In the case of the second technology [10], consisting of the preliminary induction heating of the charge to a temperature over 1000 °C, which is directly followed by forward extrusion [13] (first step) and then forging (second step), the microstructure test results show that elements manufactured by means of this method have a uniform microstructure and excellent mechanical properties [14]. Additionally, the supplied bars have bigger diameters, as well as a smaller tolerance on the diameter and a higher roughness, which lowers the price of the charge material. The first forming operation consists of extruding a roller to form the valve shank [15]. The applied lubricant is used to control the heat exchange and reduce the inter-phase friction. An advantage of extrusion is the three-axial state of stress (compression), as well as the lack of material losses, whereas the flaws of this process include high unit pressures and low tool durability [16]. After extrusion, some parts are thrown out of the first seat and transported to the second forming station. In the second operation, the valve head is forged. A sufficient amount of material to fill the forging’s cavity should be supplied, so that there are no shortages (faulty forgings) [17]. Too much material causes the formation of a flash at the spot of the joint between the upper and lower tool, causing difficulties during mechanical treatment. And so, by applying the second technology, consisting of a process of extrusion and forging for the production of valves, we obtain better mechanical properties of the manufactured component at the cost of a very low tool durability, caused by, e.g., high unit pressures on the blocking die, high process temperatures, or changeable tribological conditions [18]. In the case of producing valves, the key aspect is the durability of the forging tools, as the price of a forging strongly depends on the number of forgings produced by a given tool. Another aspect reducing tool durability is the applied valve material, especially precipitation-hardened chromium–nickel steel, with poorly soluble chromium carbides on the austenite grain boundaries, as well as simple carbides inside the grains [19]. Then, the carbides located on the grain boundaries do not dissolve, leaving their hardness at the level of that of the material as-delivered and causing, e.g., blocking of the slug forging in the die [20,21]. Additionally, chromium–nickel steel is characterized by increased adhesion to the tool, which also worsens its performance properties and accelerates tool wear [22,23]. During their work, forging tools are exposed to the operation of many destructive mechanisms, disqualifying them from further use. The most common ones include abrasive wear, adhesive wear, thermo-mechanical fatigue, plastic deformation, fatigue cracks, and oxidation [24]. The result of the wear of a forging tool is a worsened quality of the forging, which generates costs connected with frequent tool replacement as well as an increased reject rate. Thus, the biggest advantage of the analyzed process is the determination of both the causes of destructive mechanisms and the methods of their elimination [25,26]. Forging tools work under extremely difficult operation conditions, being exposed to very high pressures, intensive friction, and cyclic temperature changes, and all this shortens the period of their work [27]. The frequency of tool replacement significantly raises the costs of the forging process, prolongs the machine downtime connected with tool replacement, and increases the number of rejects caused by the more frequent process initiation on new tools. For this reason, it is very important, especially in the case of series production, to improve the durability of the tools [28]. There are a few definitions of tool durability; nonetheless, it is assumed that it is the number of cycles that can be performed by means of one tool obtaining products fulfilling the quality requirements [29]. The literature provides many methods for increasing tool durability, such as the tool material, surface layers, the improvement of working conditions, the or improvement of the forging process and operation [30]. The main methods of improving tool life include the selection of the tool material for the given process [31,32,33]; its adequate thermal [34] and thermo-chemical treatment [35,36]; hybrid techniques [37,38], with the use of physical vapor deposition (PVD) coatings [39,40]; as well as the optimization of the die shape and tool set construction [41]. To analyze industrial metal-forming processes, numerical modeling and simulations based on the FEM (Finite Element Method) or FVM (Finite Volume Method) [42,43] are very often used, because from numerical modeling results, we can obtain a lot of very important and hard-to-determine (through experience) technological parameters, as well as different physical values/variables. Currently, such computational packages (Forge, Simufact, QForm) allow for quick analyses of the entire industrial process, e.g., determining the distribution of temperatures, stresses, forging forces, flow errors of the deformed material, and many other technological aspects [44,45]. Furthermore, by using new, special functions verified in industrial conditions, they even allow for the detection of flaws, like overlapping folds or trapped “pressure pockets”, and compare numerical results and z-nominal CAD models [46].

The selection of the proper method depends on the occurring destructive mechanisms, as well as their intensity and area of occurrence on the tool. Furthermore, analyses of forging tool wear show that, in many cases, the best solution is the application of a few methods simultaneously to effectively improve the performance properties. Nevertheless, implementing particular methods of durability improvement may cause a change in the operational state, that is, the removal of some destructive mechanisms, which are, however, replaced by other new, dominating mechanisms. It is also crucial to consider the economical aspect, as the introduction of tool improvement methods is relatively expensive, and the time of waiting for the results is long because they requires confirmation with a larger number of tools. Thus, many forges often cannot afford such improvements and changes. For this reason, in the first place, based on a complex analysis of the given process as well as experiments, we should implement those methods which are verified and have the highest probability of improving durability in respect of the ease and costs of implementation of a given solution into industrial conditions. Such confirmed and relatively inexpensive methods of durability improvement include changing the tool material, surface engineering techniques, and technological changes.

The authors of this study, throughout their many years of R&D work, have analyzed numerous greater or lesser tool life improvement methods [47], and additionally, each forging process, even its particular operations, should be treated individually, which also determines the selection of durability improvement methods [48,49,50]. This article presents results regarding the possibility of increasing the durability of tools by using one of the easiest to evaluate methods, the obtained results of which are also easy to interpret, i.e., the method of increasing the durability of forging punches, which involves the use of an alternative tool material. This approach has been confirmed in many works [51,52,53], as it is one of the cheapest and relatively easy-to-analyze methods of increasing the durability of forging tools.

## 2. Test Subject and Methods

**Figure 1:**
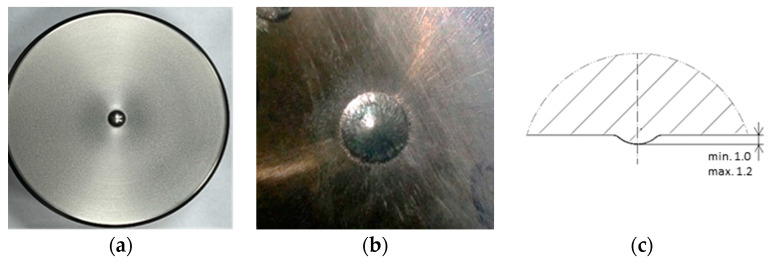
View of (**a**) the punch front surface, (**b**) the calotte, (**c**) and the requirements for the calotte according to the technical drawing (cross section).

The subject of this investigation is a detailed analysis of the forging tools (punches) applied during the manufacture of valve forgings, together with the proposal of a durability increase through the use of alternative tool materials. The process of forming a valve forging for motor truck engines takes place in two stages. The first operation involves hot co-extrusion, followed by the forging of a valve disk. During the forging process, the tool that undergoes premature wear in the first operation is the die, whereas, in the second operation, the fastest wear is observed in the case of the punch, especially its area responsible for forming the calotte on the front surface of the valve disk. Thus, the process selected for the analysis includes two pairs of tools, dies and punches, and for each of them, based on the real production process, the average durability was determined, at which point we should closely observe the given tool, as it can begin producing defected forgings. In the case of punches used in operation II, the average durability is about 1500 forgings. For this reason, a detailed analysis was performed on the forging punches used in operation II in closed dies, in a hot process (Figure 1).

The punches used in the production process are made from tool steel 32CrMoV12-28 (1.2365), after thermal treatment to a hardness of 52–53 HRC. The tools are subjected to standard thermal treatment, i.e., hardening and two-fold tempering, to obtain the assumed hardness. They are not nitrided due to their multiple use after operation through mechanical treatment, consisting of lowering their height and preparing a new engraving. In the case of punches, the key element is their front surface, especially the so-called calotte, which forms a spherical cavity in the upper surface of the valve head. One of the functions of the calotte is the proper volume. This is connected with the gas compression in the combustion chamber, as this is where the mixing and directing of the air stream during combustion takes place. Another function is the technological exit of the tool during the rolling of the valve’s front surface. For this reason, during the forging of valves, it is very important that the operators control the calotte’s depth and visually evaluate its shape—these are the two factors that decide the punch’s durability.

The current technological flow is as follows: The workpiece material in the form of a cylinder is heated in an induction furnace to a temperature of 1040–1060 °C, while the working temperature of the tools is approximately 200 °C (heating with hot waste material). The forging process is carried out on an eccentric press with a maximum pressure of 700 tons, and due to the simultaneous occurrence of two operations in one press movement, the total pressure used in both operations is over 300 tons. In the industrial process, lubrication in the first operation of the analyzed tools (dies) is carried out using a special ring (flat sleeve) mounted in the die housing. In the second operation, lubrication is performed with nozzles installed on the manipulator carrying the forgings. The stamps are not lubricated and not cooled. The grease is a mixture of graphite and oil in a 1:12 proportion, and is fed automatically by a special lubrication device. During the forging process, in order to assess the condition of the tools, the so-called control forgings, according to the control card, are collected every 300 pieces. The operator then performs a visual inspection, based on which he assesses the quality of the forging and the presence of any surface defects. In addition, he measures two key geometric features: the depth of the calotte in the outer surface of the forging plate and the radial/axial runout of the forging leg.

During the analysis—in particular, the durability of the stamps from the 2nd operation—the following scientific techniques were applied:

- Macroscopic tests combined with a measurement of the wear/allowance on the tool working surface through 3D scanning with a laser scanner and a comparison of the scan geometry with the CAD model;
- Numerical simulations based on the Finite Element Method carried out in the Forge 3.0 NxT program for the presently realized technology, as well as other tribological conditions (change in temperature and friction) of the forging process, which can occur under industrial conditions;
- Microstructural tests performed in the surface layer of the tool’s cross-section by means of the light microscopy method after its initial etching;
- Observations of the changes taking place on the working surface using a scanning electron microscope (SEM);
- Microhardness measurements on the cross-section considering the function of the distance from the surface by means of an LECO microhardness tester;
- Other methods and research techniques.

## 3. Results and Discussion

The investigations were divided into a few stages, the first one being a complex analysis of the currently realized technology, followed by the application of numerical modelling to determine the key parameters and physical quantities for a more thorough process analysis. The second stage involved attempts at using alternative materials for the punches in order to examine the possibilities for increasing the time of their operation.

### 3.1. Analysis of Durability in the Present Technology

**Figure 2:**
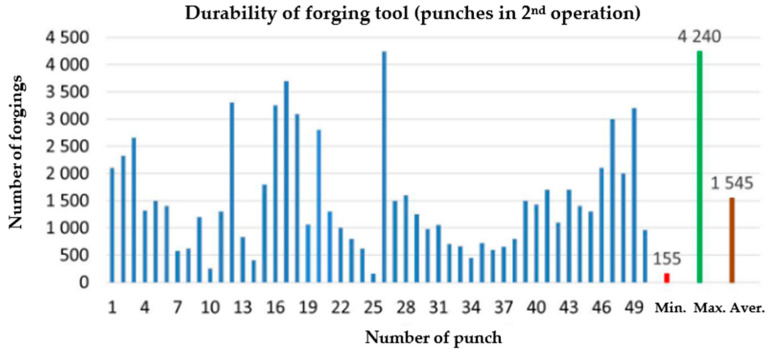
Diagram of the average durability of punches used in operation II (data from 2020).

The analyzed production process applies the second forging technology mentioned in the state of the art, which consists of extruding a pear-shaped forging in the first operation. In turn, the second operation is a process of hot forging in closed dies. Despite the obvious advantages in terms of the quality and performance properties of forgings obtained using this technology, the latter is difficult to master for many reasons resulting from the process and technological conditionings. The biggest problem are high pressures in the tools as well as cyclic thermo-mechanical loads, which cause problems with the hardness of the punches and, thus, the production of defective austenitic steel forgings. The low tool durability from this technology also results from the difficulties in the forming of steel with a high nickel and chromium content due to increased adhesion to the substrate of a tool made of 1.2365 tool steel, the charge material being made of this steel (nickel content over 20%, chromium content 15–20%). A big problem is also the difficulty in dissolving hard carbides in the charge material, resulting from insufficient heating/overheating of the charge in the whole volume. The hard working conditions of the forging tools, as well as high pressures and temperatures, cause increased wear of the punch’s calotte, mainly as a result of abrasive wear and plastic deformation. Based on the data collected from real production, the average minimal and maximal hardness values for the punch in operation II were determined (Figure 2).

To that end, 50 consecutive punches were collected from the current production after the end of their operation time. The minimal hardness value is at the level of 155 forgings, the maximal is 4240 forgings, and the average equals 1545 pieces. As we can observe, the wear of the tools is very unstable and, in effect, affects both the product quality and the press efficiency. Thus, from the whole series, representative tools were selected for testing, which had worked over different, increasing numbers of forgings to perform a global analysis of these tools’ wear.

**Figure 3:**
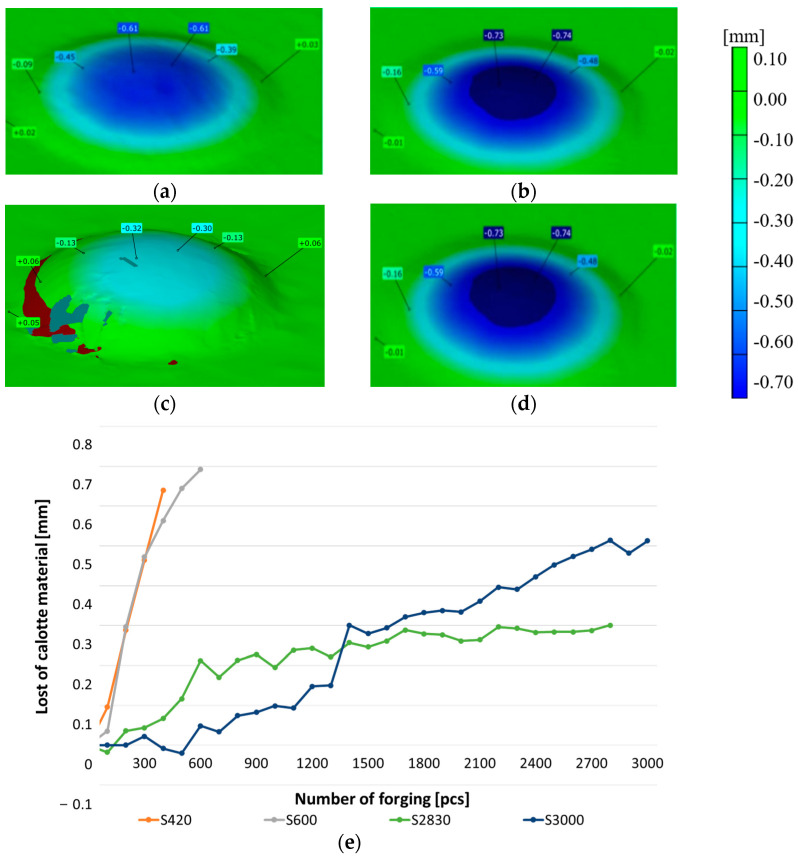
A color map of scans for the selected calottes (**a**) after 420 forgings, (**b**) after 600 forgings, (**c**) after 2830 forgings, (**d**) and after 3000 forgings, and (**e**) a diagram of the calotte’s height loss obtained based on the reverse scanning method for cyclically collected forgings.

For the macroscopic tests, alongside the typical tools used for macroscopic observations, 3D scanning techniques were also applied, which are successfully used for the evaluation of the wear of removed tools, mainly owing to their easy analysis and interpretation of results. The obtained results in the form of tool scans, especially those of the key punch element, the calotte, were compared with the tool’s CAD model. On this basis, a color map of deviation was created, based on which we can determine the areas of the greatest wear and analyze the changes during the whole operation period for a given tool. The representative results in Figure 3a show scans of the calottes on punches removed from the production process. For a better understanding of the mechanism of losing height from the punch, additional samples were collected, every 100th item, on which scans were performed by means of the reverse scanning method (Figure 3b).

As we can see, the differences in the tool durability are very big, whereas the material loss is identical. Both the punch which worked over 420 forgings and that which produced 3000 forgings lost a similar height value, i.e., about 0.6 mm. The analysis of the presented diagram after the first 100 forgings shows the direction in which the material loss curve will be positioned. For punches with a low durability, the loss of the calotte material runs in a snowball manner, and with each next collected forging, there is a clear loss of the calotte’s height. In turn, for punches whose durability was much lower, the loss of the dimension is uniform (steady). At the same time, based on the obtained scans, we can state that they were prematurely removed from the process, as the S2830 punch had lost its height to 0.3 mm, while S3000 has lost height to 0.5 mm, with an acceptable dimension loss of 0.6 mm.

### 3.2. Microscopic Observations

**Figure 4:**
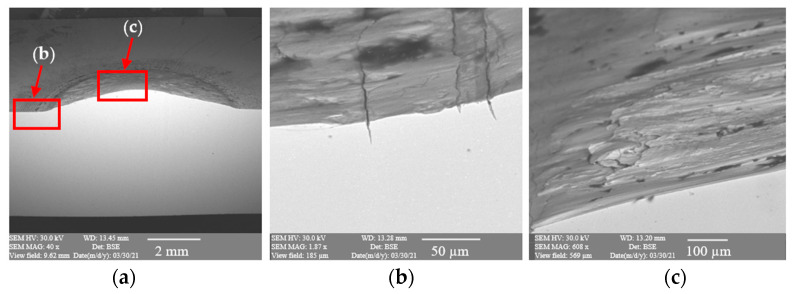
Results of microscopic observations SEM for a punch which worked over 400 forgings: (**a**) marked area of the microstructure and microhardness analysis, (**b**) calotte base, (**c**) calotte.

**Figure 5:**
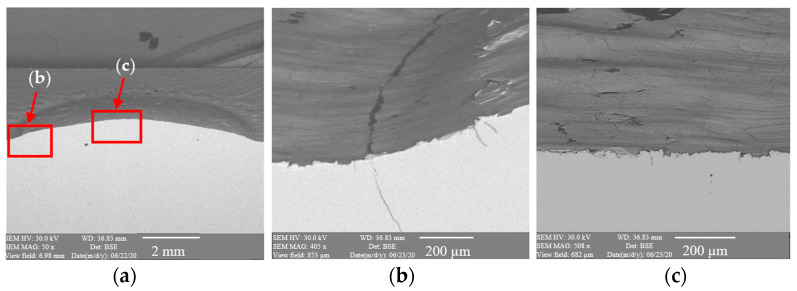
Results of microscopic observations SEM for a punch which worked over 2600 forgings: (**a**) marked area of the microstructure and microhardness analysis, (**b**) calotte base, (**c**) calotte.

To better understand the destructive mechanisms occurring on the punches during their operation, microscopic tests with the use of light microscopy methods and a scanning electron microscope were conducted on the microstructures of punches made of 32CrMoV12-28. To present the obtained results, examples of two punches were used. One, which produced 400 forgings, represented a group of tools where the durability loss proceeded in the snowball manner. The other, in turn, came from a group of tools in which the durability loss proceeded more slowly, and it was removed from the process after producing 2660 forgings. In a comparison of the photographs of punches taken on a scanning electron microscope (Figure 4 and Figure 5), we can see, at the base of the calotte, fatigue cracks in the material on both punches. The depth of the cracks depends on the number of produced forgings. The punch which made 400 forgings has a crack of about 25 μm (Figure 4b), whereas that which worked over 2660 forgings has a crack over 200 μm deep (Figure 5b).

**Figure 6:**
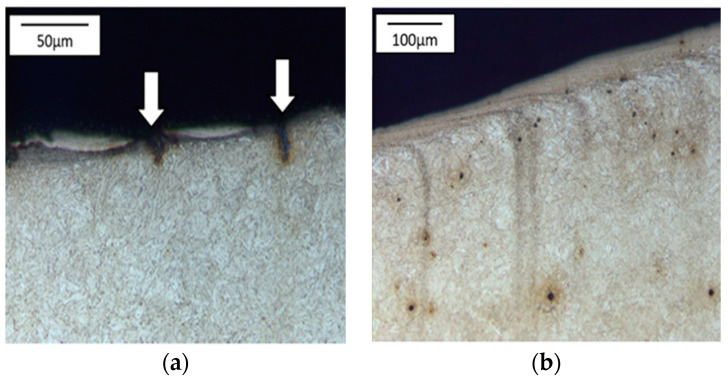
Microstructure of the punch material which worked over 400 forgings: (**a**) base of the calotte (the arrows indicate cracks and corrosion of the surface layer), (**b**) calotte. Light microscopy, etched state.

The cracks are a result of a changeable load as well as varying temperature fields, which were observed in the industrial process and proven through numerical simulations. In the calotte area, on both punches, we can see plastic deformations and material losses, more numerous in the case of the S2660 punch. The nominal material used for the punch is 32CrMoV12-28 tool steel. Based on the performed analysis, it can be stated that the calotte on the punch undergoes plastic deformation due to the progressing tempering of the tool material. The evaluation of the microstructure made by means of a light microscope on the S400 punch (Figure 6) shows the strong adhesion of the charge material to the tool material; we can additionally see minor fatigue cracks in the material, marked in Figure 6a.

The tool’s microstructure is highly tempered martensite. In the calotte area, we can observe small carbide bands (Figure 6b), which also undergo deformation and become oriented in the surface layer in the direction of the material flow.

**Figure 7:**
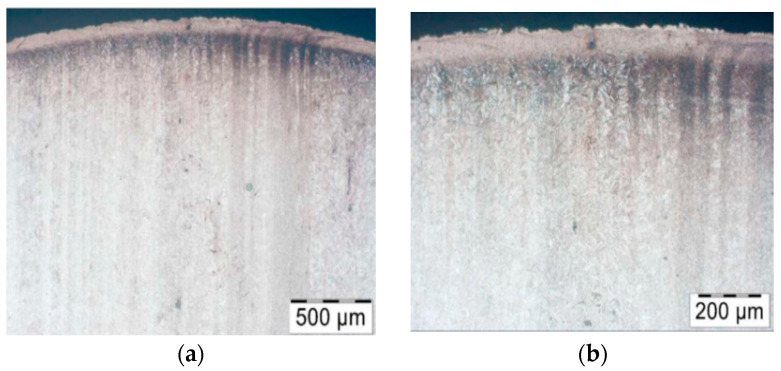

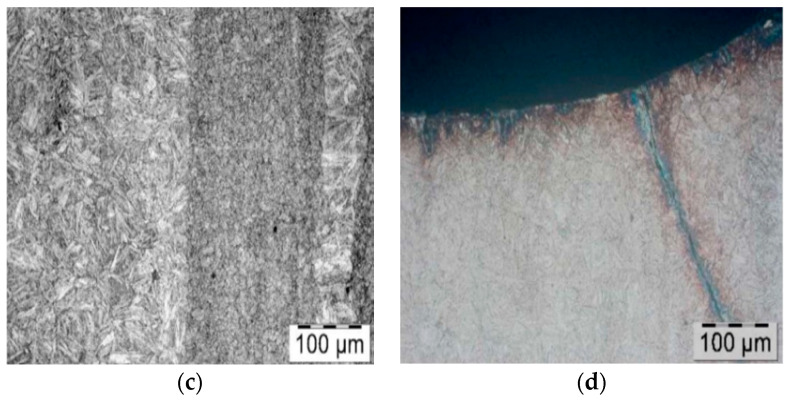
Microstructure of the punch material which worker over 2660 forgings: (**a**) calotte, (**b**) calotte magnification, (**c**) fine dispersive secondary carbides, (**d**) calotte base. Light microscopy, etched state.

The S2660 punch (Figure 7) also has bands with a high amount of fine dispersive carbides in its microstructure. On the top of the calotte, we can observe a white layer with stuck fragments of the forging material, whereas, at its base, there is a fatigue crack (Figure 7d).

### 3.3. Microhardness Measurements

**Figure 8:**
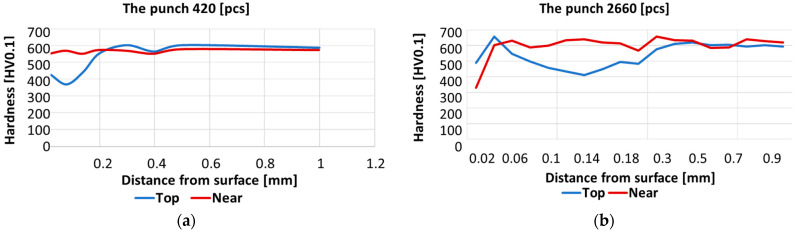
Hardness distributions of the punch cross-section: (**a**) S420, (**b**) S2660.

The hardness distributions were determined with the Vickers method in the areas marked in Figure 4 and Figure 5 from the head surface of the punch into the material. The results are presented in Figure 8.

In the case of the S420 punch, the peak of the calotte became tempered over 400 HV0.1, whereas the calotte base preserved the tool material hardness, i.e., about 600 HV0.1. The S2660 punch at the calotte base underwent tempering over 400 HV0.1, while at the calotte peak, we can see an increase in hardness to 650 HV0.1, which is probably caused by the presence of a white layer and, despite plastic deformation, the layer still exhibits high hardness. Further deeper into the material, we observe tempering even to the depth of 0.3–0.4 mm.

## 4. Numerical Modelling

To more precisely determine the main factors influencing the forging, a numerical analysis with different variants of technological parameters was conducted for the current technology (for the second operation). Conditions consistent with those of the industrial process were assumed, and then, the tribological conditions were slightly changed in respect of the nominal technology. On this basis, a thermo-mechanical model was elaborated, which was used for the analysis of the tool wear. In the simulation of the forging process, an axisymmetric model with deformable tools was applied. It was assumed that these are “purely elastic” bodies, with a Young’s modulus of 200 GPa and a Poisson number of 0.3. In the numerical modeling, the friction model according to Treska was used as one of the available friction models in the utilized FEM software (Datafile Forge 3v75), which is commonly used to simulate forging processes [54]:

(1) $$\vec{\tau }=\bar{m}\frac{\sigma_{o}}{\sqrt{3}}\;\frac{∆\vec{v}}{∆\vec{v}}$$

where

m—is friction factor in range from 0 to 1;

σ_0_—von Misses stress;

Δv—relative velocity in contact.

**Figure 9:**
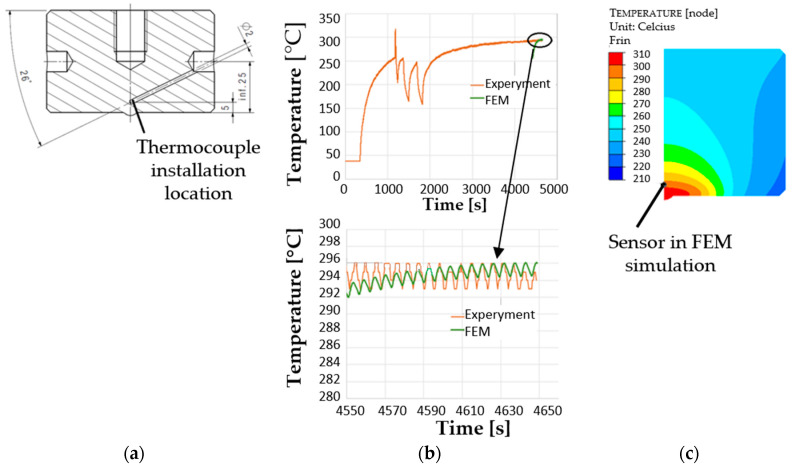
View of (**a**) a draft of the punch with a drilled opening for the thermocouple, (**b**) the real temperature measurement on the punch, and (**c**) the temperature distribution after 41 cycles with the marked area of the temperature measurement.

The assumed punch working temperature was 250 °C. For the other tools, the temperature was 150 °C, and that of the environment was approximately 30 °C. The temperature assumed for the punch in the numerical simulations was verified in the real production process. For that purpose, a punch was prepared, with a drilled opening for the thermocouple by the calotte (Figure 9a). The assumed heat exchange coefficients between the forging and the punch were 1.5 kW/m^2^ K. As the tool material, steel X0CrMoV5-1/1.2344 from the Forge database was selected. The initial temperature of forging was 1050 °C.

**Figure 10:**
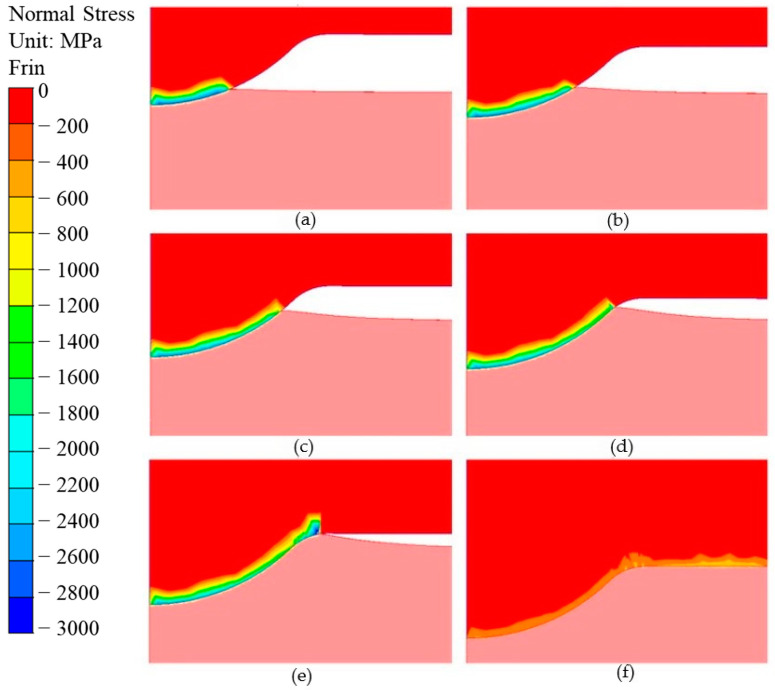
Distributions of normal pressures on the calotte surface for a cavity of (**a**) 0.3 mm, (**b**) 0.6 mm, (**c**) 0.9 mm, (**d**) 1.2 mm, (**e**) the whole calotte, and (**f**) full contact of the punch with the forging material.

Firstly, an analysis of the stresses present during forging on the calotte—the key element of the punch—was performed. The normal pressure distribution on the calotte surface for selected heights of the punch’s submersion into the material is shown in Figure 10. The analysis results confirmed that the highest stresses and normal pressures are present exactly on the calotte, and their high values can cause the formation of cracks and plastic deformations, leading to the flattening of the calotte. We can notice that, in the first operation of forging a valve, on the contact surface of the calotte with the forging material, the normal pressures reach very high values of as much as 1800 MPa. Only after the punch working surface reaches full contact with the forging head does the stress value in the calotte decrease—to about 400–500 MPa—and distributes uniformly on the contact surface of the punch with the forging.

**Figure 11:**
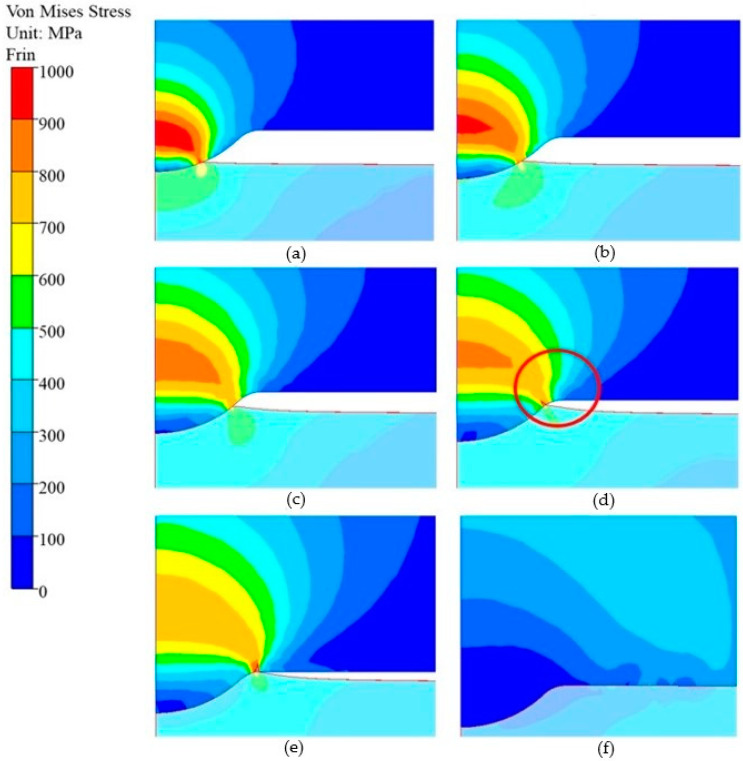
Distributions of normal stresses on the calotte surface for a cavity of (**a**) 0.3 mm, (**b**) 0.6 mm, (**c**) 0.9 mm, (**d**) 1.2 mm, (**e**) the whole calotte, (**f**) and full contact for the punch with the forging material.

The reduced stress distribution according to the Huber–Mises hypothesis inside the punch in the calotte area, during the formation of the valve, is presented in Figure 11. As in the case of normal stresses, the highest reduced stresses in the calotte area are present in the first stage of forging the valve and, locally, on the calotte circumference, they equal up to 1000 MPa. After the whole working surface of the punch comes into contact with the forging head, the stresses decrease to about 50–100 MPa and distribute more uniformly. The stress concentration marked in Figure 11d, locally reaching 1000 MPa, can cause the formation of cracks near the calotte base on the punch, which was observed during the analysis using microscopes (Figure 5b and Figure 6b).

The presented results of numerical modeling should be treated as a nominal process that is assumed based on the technology (what should be observed if the assumed conditions are fulfilled).

Additionally, due to the relatively low repeatability and stability of the industrial process, numerical modelling was also performed for a few other variants of the realization of a virtual forging process, which can take place under industrial conditions. Such a decision was made because the performed observations demonstrated that such a situation can occur and can be connected with difficulties in controlling the process parameters or the insufficient mastering of the difficult technology of forging in closed dies. This is also justified by the fact that the tests and trials conducted under industrial conditions for different variants could cause significant difficulties, as well as shutdowns, in the current production, and thus bring economic losses. The obtained results for the multi-variant simulations show what can happen if the assumed conditions are different, e.g., due to improperly implemented technology or other unpredictable situations. To determine the limit values of the influence of temperature and tribological conditions on the course of the industrial forging process, five different variants were assumed, which, in the authors’ opinion, can significantly contribute to the premature wear of forging punches:

I. A nominal process assumed by the current technology, i.e., workpiece material temperature: 1050 °C; the punch and other tools’ temperature: 250 °C; and the friction factor assumed to be the Tresca factor: m = 0.2;
II. The input material temperature is decreased to 950 °C, with the other technological parameters assumed as for the process according to the current technology (variant I);
III. The punches and the other forging tools’ temperature is raised up to 300 °C, with the other parameters of technology as in variant I;
IV. The conditions of the forging process are adopted in accordance with the existing technology, but with the friction coefficient increased to m = 0.6;
V. The temperature of the input material is raised to 1150 °C, with the friction coefficient according to Tresca increased to m = 0.6.

**Figure 12:**
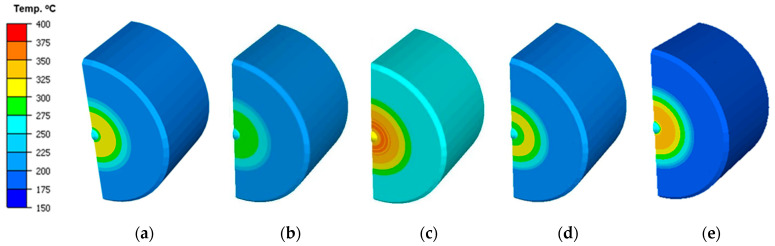
FEM results with the temperature field distributions on the punch for the selected process variants: (**a**) nominal variants I, (**b**) II, (**c**) III, (**d**) IV, and (**e**) V.

An analysis of the distribution of the temperature fields, forging force patterns, and total consumption was carried out for the five selected representative variants that may occur in the industrial process, referring the results to the currently realized technology, i.e., variant II. Figure 12 presents the field of temperature distributions on the punch in contact with the forging for the particular variants (for half of the punch in an axisymmetrical system) in the final phase of the forging process. By analyzing the distribution of the temperature field, it can be observed that for a punch with an increased initial temperature, the temperature field distribution is entirely different (higher temperature in the whole volume) than in the case of the other variants.

**Figure 13:**
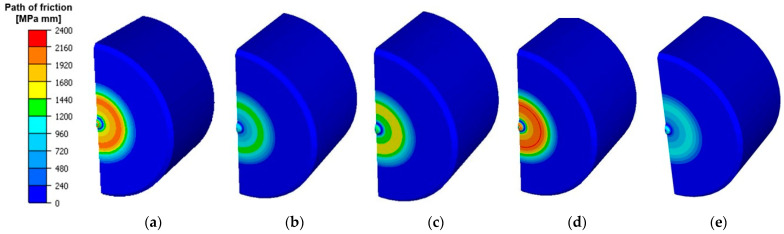
The FE results of global abrasive wear of the chosen simulation variants: (**a**) I—nominal technology, (**b**) II, (**c**) III, (**d**) IV, and (**e**) V.

In turn, for the punch with increased friction (IV), which, as a consequence, causes a longer contact time, we can observe a slightly elevated temperature in the calotte and its vicinity. In the case of variant V, we can notice that the area from the calotte to the spot of intensive abrasive wear (traces of a circle) has the highest temperature, approximately 250 °C, compared to the other tools, with the obvious exception of variant III (a punch with a higher initial temperature). Moreover, the performed tests of the punch temperature in the industrial process demonstrated that, at a distance of few millimeters from the calotte surface, the temperature is higher than that in the currently technology, and thus also in numerical modelling, as it equals 280 °C. Based on this, we can state that the technology realized so far still requires many analyses and tests so that it is possible to collected additional information, based on which proper remedial actions can be taken. Figure 13 presents the numerical simulation results with the abrasive wear distribution. The obtained results show that the highest wear occurred for the punch with the temperature elevated to 300 °C. Unfortunately, this can cause a lowered yield point in the industrial process, both for the tool material and the forging material, due to a larger amount of supplied heat. This, in turn, allows for the better flow of the forging material along the tool’s working cavity, but also causes its increased wear.

For the other analyzed variants, the distributions of wear are similar. Only in the case of punches with increased factors of friction is the abrasive wear slightly smaller, which can be explained by the hindered material flow along the punch, which, in turn, translates to lower wear. The highest wear values are observed at a certain distance from the punch axis in a radial direction (towards the outside of the material), in the area where increased abrasive wear and adhesion were observed. However, due to the relatively high costs of the proceedings, it is difficult to perform long-term tests under industrial conditions for the chosen, most probable variants, other than using the current technology, i.e., the one assumed as the proper technology for producing valve forgings. The presented supplementary simulation test results (multi-variant FEM simulations) allowed us to obtain a lot of information and parameters that would be difficult to achieve in the analysis of industrial technology, and the detailed results showed that, for the forging punches used in operation II, it is justifiable to apply one of the selected durability-improving methods. Based on this, we can also state that even small changes to the key tribological parameters, such as the temperature of the charge and the tool as well as lubrication, significantly affect the process and thus also the tool durability, and can cause premature tool wear. For this reason, owing to its high effectiveness and ease in analyzing the introduced changes, a decision was made to apply alternative tool materials as the method of improving the durability of forging instrumentation. For this purpose, specialized materials belonging to the group of tool steels, dedicated to forging punches used in forging processes on a press, were selected.

## 5. Selection of Hot-Operation Steel for the Punch

The nominal material used for the punch was 32CrMoV12-28 tool steel (1.2365 according to DIN). Based on the performed analysis, it was established that the calotte on the punch undergoes plastic deformation due to the progressing tempering of the tool material. Because of this, other tool materials, whose tempering temperature is much higher than the one originally applied, were analyzed. Thus, in order to improve the life of the punches in the second operation, steels W360, S600, and S705 were selected, whose chemical compositions are presented in Table 1 according to the technical specification provided by the producers.

**Figure 14:**
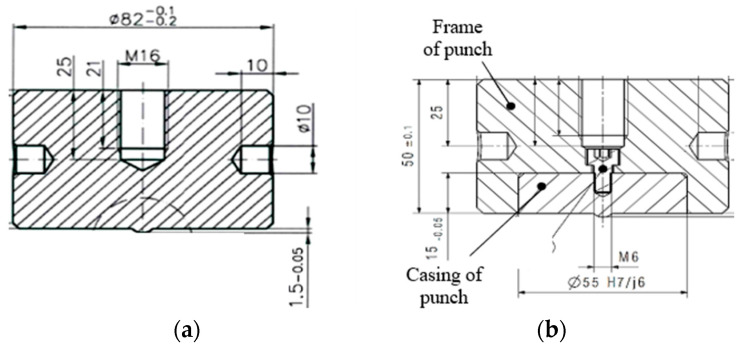
A drawing of a punch: (**a**) monolithic, (**b**) combined (an insert screwed into a casing).

W360 is a hot-work steel which is characterized by high hardness, abrasive resistance, and strength. In turn, steels S600 and S705 are high-speed steels which exhibit high tempering resistance, high hardness at elevated temperatures, and abrasive resistance. The materials listed in Table 1 were used to prepare monolithic tools from W360, whereas the high-speed steels were applied to make a combined tool, i.e., a high-speed steel insert and a 32CrMoV12-28 casing (Figure 14).

**Figure 15:**
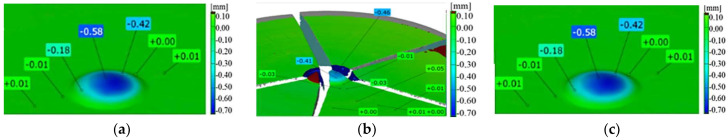
A scan image of the calotte on the tools made from (**a**) W360, (**b**) S600, and (**c**) S705.

This is caused by both the high costs of preparing the tool and the loads coming from the press being taken over by the material of the punch casing. From each steel grade, three tools were made and the punches were introduced into the production process. From each group of tools, one representative punch was selected for the analysis of the wear mechanisms. Initially, scans of the front surface of the punch were made and the shape of the calotte was analyzed (Figure 15), precisely its height.

The average deformation on each tool equaled 0.5–0.6 mm. After a loss in the calotte height, the punch was removed from process. In the case of the punches made of steel S600, the tools cracked before reaching the critical calotte depth, which proves the high brittleness of the material.

**Figure 16:**
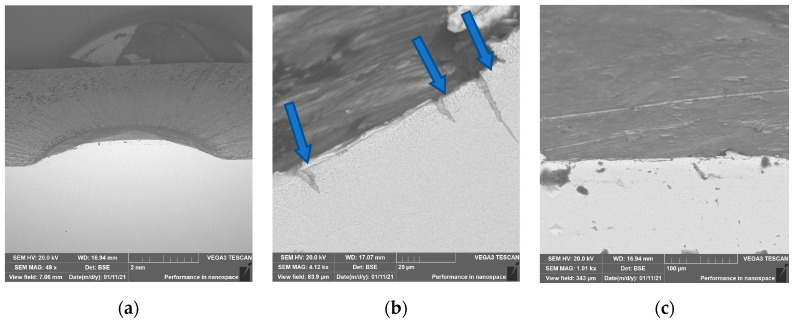
SEM images in the subsurface area of the cross-section of the W360 punch: (**a**) the calotte area, (**b**) the calotte slope (blue arrows show cracks), (**c**) the calotte peak for this punch.

The image obtained using a scanning electron microscope shows the punch made of W360 after producing 1480 forgings (Figure 16). The tool had numerous fatigue cracks on the slope of the calotte, proving a long performance time, as well as plastic deformations and spalling on the calotte’s peak.

**Figure 17:**
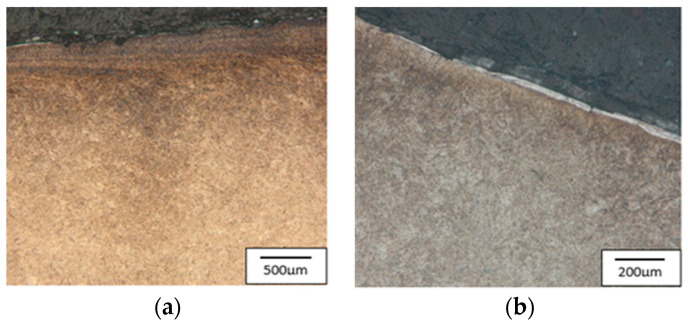
Microstructure of the punch made from W360: (**a**) the calotte peak, (**b**) the calotte slope. Light microscopy, etched state.

Figure 17 shows the microstructure of the tool made of steel W360 in the surface-layer area. Both on the calotte’s peak and slope, a large material work-hardening can be visible—a fine grain and a characteristic material flow in the direction of the calotte’s formation (Figure 17a). On the slope, we can notice stick-ons of the charge material as well as fine cracks (Figure 17b).

**Figure 18:**
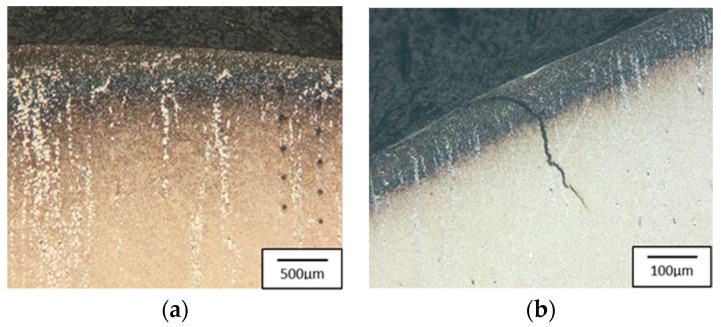
Microstructure of the punch made of S600: (**a**) the calotte peak, (**b**) the calotte slope. Light microscopy, etched state.

**Figure 19:**
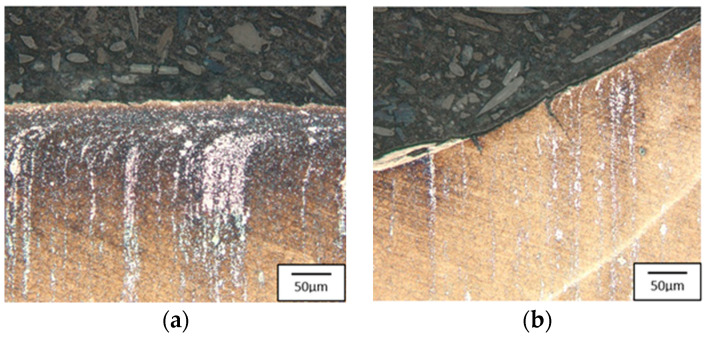
Microstructure of the punch made of S705: (**a**) the calotte peak, (**b**) the calotte slope. Light microscopy, etched state.

The microstructure of steel W360 is highly tempered martensite, with visible trace amounts of retained austenite—light-colored areas. The consecutively analyzed tools were made from high-speed steel, S600 (Figure 18) and S705 (Figure 19). A typical structure for this steel is cryptoacicular martensite, as well as M6C and MC carbides.

**Figure 20:**
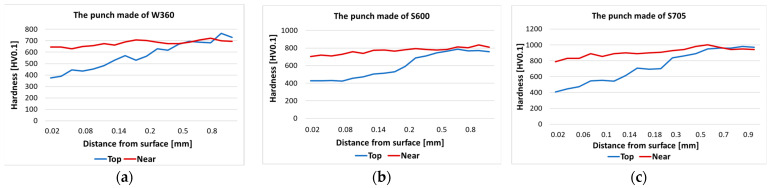
Microhardness distribution on the punch: (**a**) W360, (**b**) S600, (**c**) S705.

In both cases, we can see a clear band-like orientation of the carbides (Figure 16 and Figure 17). The punch made of steel S600 etched more intensively both on the edge and the slope of the calotte, whereas for the tool made from steel S705, this was only at the top of the calotte. The more intensive etching is connected with the disintegration of the martensite and the intensification of the precipitation processes. On the slope of the S600 tool, we can see a clear crack and minor stick-ons of the forging material. For the S705 punch, there is a visible flow of carbides by the tool surface, and at the base of the calotte slope, we can see minor cracks as well as a layer of stuck forging material. Figure 20 shows the results from the microhardness measurement made on the peak and the slope of the calotte into the tool material in the areas marked in Figure 4 and Figure 5.

An analysis of the hardness curves shows that, on each used punch, on the top of the calotte, material tempering takes place to a hardness of about 400 HV, at a depth of up to 0.5 mm. At the calotte’s base, the material does not undergo tempering, maintaining its hardness before the operation.

Table 2 presents the average durability of each of the three tool materials selected for the tests.

The obtained complex results of the conducted tests demonstrate that the alternative materials from the group of tool steels applied for the punch do not confirm the target application in the industrial process, as none of the tools obtained the assumed results and the average durabilities are lower than those for the standard material used so far for the tools.

### Application of Punch Inserts Made of Sintered Carbide

**Figure 21:**
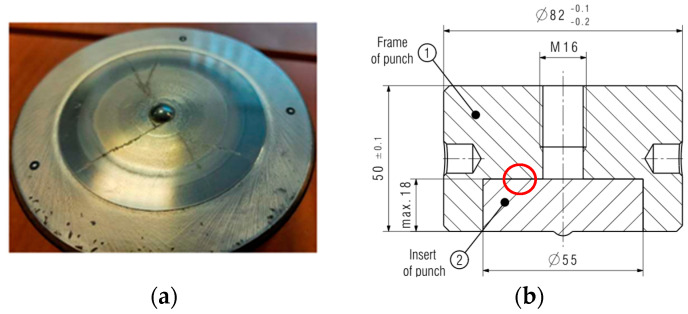
View of (**a**) a cracked carbide insert S30, and (**b**) the construction of the S30 punch with a carbide insert, with the marked constructional notch.

For this reason, a decision was made to apply sintered carbide as the tool material. And so, the following method of increasing tool durability was the use of an insert made of sintered carbides, which are characterized by high temperature resistance. Additionally, the high values of the Young’s modulus and hardness of sintered carbides ensures low susceptibility to plastic deformations, which makes this material possibly effective in ensuring the elevated durability of punches. In the first attempt at increasing the punch durability, an insert made of sintered carbide with a hardness of 1340 HV was used (S30). The design of the tool is shown in Figure 21b. Three tools were made for testing, the average durability of which was 9000 pieces. The tools were withdrawn from the process because they broke or suffered a collision caused by the incorrect placement of the blank in the forging socket. However, the cracks could have come from the notch that formed under the thread (marked in Figure 21b by rec circle), near the carbide surface. The stresses that led to it probably accumulated there to break the punch.

**Figure 22:**
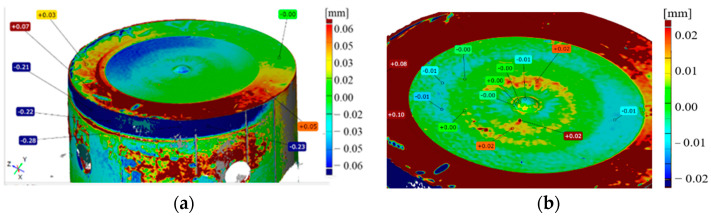
Scan of a tool with an S30 carbide insert: (**a**) the entire tool, (**b**) the area of the punch face.

Analyzing the scans of the tools (Figure 22), it can be seen that the calotte was not deformed, while the working surface of the carbide insert was not located in the upper plane of the punch holder.

**Figure 23:**
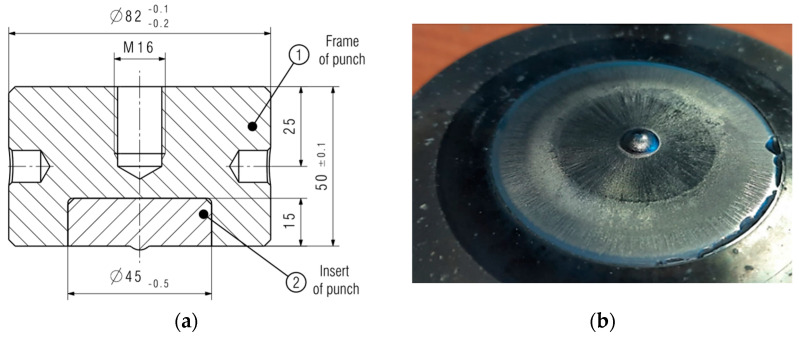
View of (**a**) the construction of a punch with carbide insert S79, and (**b**) the S79 punch after the collision.

The lack of wear mechanisms in the calotte in the case of punches from the first trial caused a decision to change the insert construction and to introduce the tools into the process again. Design changes were made by moving the end of the threaded hole above the carbide insert mounting; the design notch was eliminated (Figure 23).

**Figure 24:**
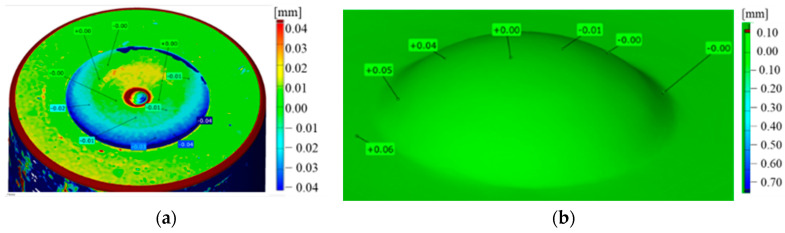
Scan of a tool with an S79 carbide insert: (**a**) the entire tool, (**b**) the cap area.

In the new technology, the carbide insert was press-fitted according to the tool supplier’s guidelines. The carbide used had a hardness of 1600 HV (S79); three pieces were prepared for testing, and their durability was 6600 pieces. By analyzing the scans presented in Figure 24, it can be seen that the cap remained unchanged. However, the material loss was located on the diameter of the carbide insert.

The tools were damaged due to chipping, both in the area of the cap and in the insert diameter, and due to a collision of the tools resulting from an incorrect arrangement of forgings in the second socket.

**Figure 25:**
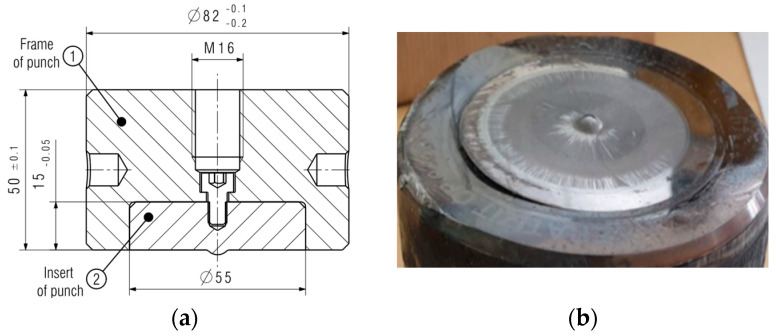
View of (**a**) the construction of the punch with an S91 carbide insert, and (**b**) the S91 punch after collision.

A third type of tool, made by another supplier, was introduced to compare wear mechanisms. The grade of sintered carbide was proposed by the manufacturer, while the design and assembly were developed by the authors. By shifting the end of the opening under the screw thread above the mounted carbide insert, the constructional notch was eliminated (Figure 23). There was also a change in the calotte height, i.e., from 1.5 mm to 1.2 mm, which is connected with the more restrictive production requirements (Figure 25a). In turn, removing the punch from the process took place as a result of a collision of the tools in the second seat, which caused the crushing of the carbide (Figure 25b).

**Figure 26:**
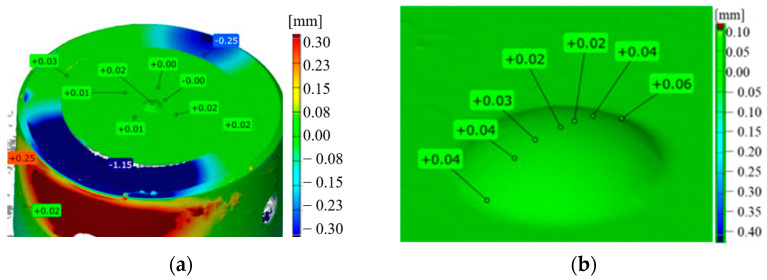
A scan image of a tool with carbide insert S91: (**a**) the whole tool, (**b**) the calotte area.

As in the case of the previous tools, the calotte did not become damaged and the punch produced as many as 20,000 forgings. This is visible in both scans (Figure 26).

**Figure 27:**
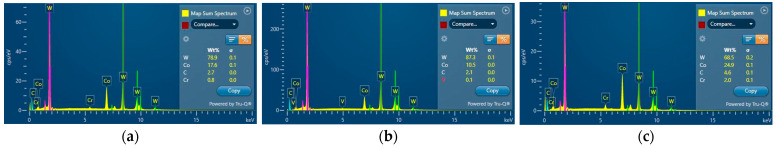
Results of the quantitative EDS analysis for sintered carbides for punch inserts: (**a**) S30, (**b**) S79, and (**c**) S91.

For a more thorough analysis of the causes of the cracking of inserts made of different sintered carbides, material tests were conducted, which made it possible to identify the chemical compositions. The tests focused on a quantitative analysis of the carbide tools. Considering the fact that the carbide properties are significantly affected by the content of the cobalt matrix, an evaluation of this fraction in the examined punches (S30, S79, and S91) was performed. For the determination of the cobalt matrix fraction, for comparison purposes, the method of digital image analysis was applied (Figure 27).

The conducted analysis showed the following chemical compositions for the three representative punches, one for each type of sintered carbide insert. Their chemical composition are shown in Table 3. The EDS analysis demonstrated the highest Co matrix fraction at the level of 24.9% for the S91 punch, 17.6% for S30, and 10.5% for S79.

**Figure 28:**
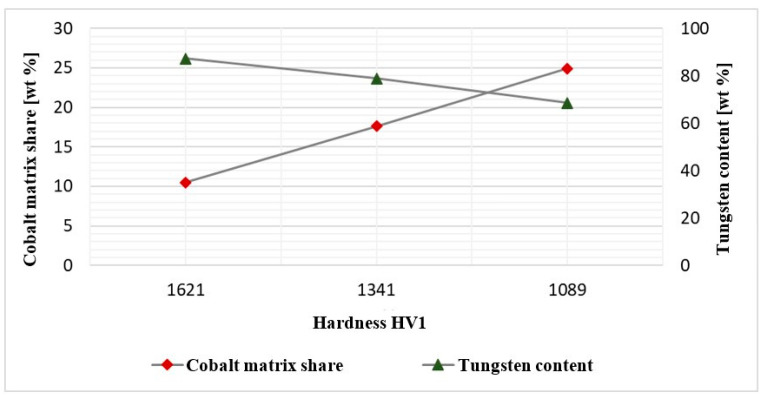
Dependence of hardness on the share of cobalt matrix and tungsten content.

This shows that, to construct the main reinforcing phase, tungsten carbide was mostly used; however, another type of reinforcing phase was also applied, i.e., Cr3C2-type chromium carbide, which is characterized by a lower hardness. The final hardness is thus affected not only by the matrix content, but also the phase composition of the carbides used in the reinforcement. We should also consider the fact that chromium exhibits high solubility in cobalt, and so we cannot exclude its presence affecting the cobalt matrix. The chromium is added at the stage of producing sintered carbides as an inhibitor of the carbide phase growth. Nevertheless, it should be clearly emphasized that, regardless of the role of chromium in the carbide material of the S30 and S91 punches, the highest tungsten content was identified in the case of the punch with the highest hardness, i.e., S79. Figure 28 shows a graph of the dependence of the average hardness on the share of the cobalt matrix and on the tungsten content. The presented results indicate that a relationship was observed between the hardness and the share of the cobalt matrix.

The presented results demonstrate a dependence between the hardness and the cobalt matrix fraction. The highest cobalt content in the S91 punch was connected with the lowest hardness, whereas the sintered carbide with the lowest cobalt content was harder. The main reason for removing all the analyzed tools from the process was the mechanical damage of the sintered carbide insert. No abrasive wear mechanisms on the punches were observed. The performed tests show that the insert made of the carbide with the lowest hardness (1089 HV) worked over the biggest number of cycles, which is why it should be subjected to further tests.

## 6. Summary and Conclusions

This study presents the results of tests, including both the tool wear and the application of alternative tool materials (for durability improvement), for tools applied in the second forging operation of producing valve forgings made of chromium–nickel steel assigned for motor truck engines. The main problem is the premature wear of the tool and, thus, its removal from the process, which is connected with the damage/defect of the key object of the punch, the calotte, which is a spherical protrusion on the front surface of the punch. Initially, a complex analysis was carried out, aided by numerical simulations for the currently realized technology, which demonstrated the following:

- The analyzed technology is a difficult and very complex forging process realized under industrial conditions, and it is crucial to properly select the optimal parameters, mainly technological, but also constructional, of the punch;
- The punch wear in the calotte area is caused by plastic deformation as a result of the tempering of the tool material;
- Another main wear mechanism taking place on the punch is the strong adhesion of the deformed forging material to the surface of the punch material, causing plastic deformations on the front surface of the valve disk;
- The numerical simulations confirmed the presence of high temperatures in the calotte area (over 300 °C), as well as very high normal stresses in the calotte base (1800 MPa) and reduced stresses (1000 MPa);
- The high normal stresses at the calotte base caused fatigue cracks, which penetrated the tool material, caused by the changing loads and temperatures;
- The performed additional multi-variant FEM simulations, together with an analysis of the results, showed that a change in the three selected parameters—the charge temperature (1040 °C) within the change scope of ±100 °C, the tool temperature (200 °C) within the change scope of ±100 °C, and the friction (m = 0.2 ± 0.6)—significantly affected the correctness of the technology;
- For this reason, in order to increase tool durability, the possibility of applying other tool steels for hot operations as well as high speed steels was analyzed. Such steels were selected whose tempering temperature is higher than that of the currently used steel and which equals over 500 °C, and whose hardness is at the level of 58–61 HRC.

Tools made of the materials with the commercial names W360, S600, and S705 were used in the tests. The performed performance analyses demonstrated the following:

- The average wear of a punch made of steel W360 equaled 1480 forgings; steel S600—850 forgings; and steel S705—810 forgings. These hardness values are lower than that of the presently used tool, i.e., WLV, for which the average hardness equaled about 1500 forgings;
- The cause of removing the tools made of the tested steels from the process was plastic deformation as a result of the tempering of the tool material. Additionally, the wear of the tools made of high-speed steels proceeded a few times faster, and the tools made of S600 became cracked;
- Due to a lack of improvement in the durability of the tool made from this steel, the authors decided to apply inserts made of sintered carbides. Three types of materials were used in the tests, which differed in the amount of the cobalt matrix and, thus, also the tool hardness. The hardness of the insert with a 10.5% cobalt matrix equaled over 1620 HV, and for the 17.6% cobalt matrix, the hardness was 1340 HV, whereas the tool with the cobalt matrix amount of 24.9% demonstrated hardness at the level of 1090 HV.

The lower the matrix content and the higher the carbide amount (reinforcement), the higher the hardness of the sintered carbide. The results of industrial tests showed the following:

- The sensitive area—the calotte—did not undergo plastic deformation, whereas both tools made a twice-as-high number of forgings than the punch used in the original production process, i.e., over 9000 forgings, whereas the S91 punch achieved over 20,000 forging;
- The tools were removed from the process due to a faulty construction of the punch (a constructional notch in the contact area of the insert with the screw thread for the mounting), as well as a collision connected with the work of the press, with no wear mechanisms observed in the calotte area.

Further tests on sintered carbides are planned, owing to their promising results. This means that the final decision on introducing the tools into the process depends on the financial costs, as one insert is over ten times more expensive than a punch made of the standard material (1.2365).

## Figures and Tables

**Table 1:** Chemical composition of steels used for punches.

Element	C (%)	Si (%)	Mn (%)	Cr (%)	Mo (%)	V (%)	W (%)	Co (%)
W360	0.50	0.20	0.25	4.50	3.00	0.60	-	-
S600	0.90	-	-	4.10	5.00	1.80	6.20	-
S705	0.95	-	-	4.10	5.00	1.90	6.20	4.80

**Table 2:** List of tools used in the research.

No.	Material for Punch	Hardness (HRC)	Average Number of Forgings (pcs.)
1	W360	58	1480 (min: 720; max: 1980)
2	S600	61	850 (min: 150; max: 1650)
3	S705	60	810 (min: 560; max: 1140)

**Table 3:** The chemical composition of the chosen sintered carbides.

Punch Number/ Elements	W (%)	Co (%)	C (%)	Cr (%)	V (%)
S30	78.9	17.6	2.7	0.8	-
S79	87.9	10.5	2.1	-	0.1
S91	68.5	24.9	4.6	2.0	-

## Data Availability

Data are contained within the article.
